# Letter to the Editor concerning “Efficacy of exercise therapy for the treatment of adolescent idiopathic scoliosis: a review of the literature” by Mordecai SC and Dabke HV (2012) Eur Spine J 21:382–389

**DOI:** 10.1007/s00586-013-2687-7

**Published:** 2013-03-16

**Authors:** Maciej Plaszewski

**Affiliations:** Institute of Physiotherapy, Warsaw University School of Physical Education, Akademicka 2, 21-500 Biala Podlaska, Poland

Dear Editor,

I have read with great interest and attention the review article by Mordecai and Dabke “Efficacy of exercise therapy for the treatment of adolescent idiopathic scoliosis: a review of the literature” [1]. The report adds a new insight into this controversial subject. However, I would like to express my concern regarding some aspects of this important paper.

As stated by the authors, they were motivated by a need to produce an unbiased review of current evidence, in opposition to previous systematic reviews published by authors engaged in rehabilitation centres specializing in treatment of patients with scoliosis. The paper covers a structure of a systematic review, and taking into account the design of included reports, I assume that this is a systematic review of observational studies. Unfortunately, Mordecai and Dabke have not followed the MOOSE methodology for reporting this type of studies [2]. I am aware that the authors did not conduct a quantitative analysis of data retrieved from reports included in their review, but most MOOSE items are credible for systematic reviews without a meta-analysis. Also, the authors have provided a wide analysis of included papers in the text, but again, it would be much easier for a reader to draw firm conclusions if an appraisal tool, e.g., STROBE [2] or the Nottingham–Ottawa Scale (NOS) had been applied. Additionally, systematic reviews considered by Mordecai and Dabke could have been appraised with the AMSTAR, a measurement tool designed to assess methodological quality of systematic reviews [2].

An example from a very corresponding field of how systematic reviews, reports intended to support clinical decision making, may differ in methodology and conclusions, and how much confusion may follow, are the two secondary analyses regarding bracing for patients with scoliosis. In 1997, Rowe et al. [3] published a meta-analysis of the efficacy of non-operative treatments for idiopathic scoliosis, including data extracted from 14 reports on bracing, selected from two unpublished studies and 37 reports identified in a single textbook. The paper confirmed the efficacy of bracing, especially when braces are worn for 23 h a day. On the other hand, a recent Cochrane review informs, based on two studies meeting inclusion criteria (with a single RCT), selected from 1,285 identified titles, that bracing is controversial, and its effectiveness remains questionable [4].

The subject of scoliosis-specific exercises brings another illustration. A pilot report regarding the effectiveness of physical exercise therapy as a single intervention for patients with scoliosis, “most often cited in support of claims that exercise cannot be used to treat scoliosis” [5], had been excluded from the systematic review by Lenssinck et al. referred also by Mordecai and Dabke due to its low scientific rigour [6].

Thus, I found a lot of interesting points and plenty of discussion from independent specialists in this significant review, but uncertainities have remained unresolved. Perhaps the subject still awaits a more quantitative analysis of both primary and secondary reports, in accordance with recognised reporting guidelines and appraisal tools.
